# ROS and ROS-Mediated Cellular Signaling

**DOI:** 10.1155/2016/4350965

**Published:** 2016-02-22

**Authors:** Jixiang Zhang, Xiaoli Wang, Vikash Vikash, Qing Ye, Dandan Wu, Yulan Liu, Weiguo Dong

**Affiliations:** ^1^Department of Gastroenterology, Renmin Hospital of Wuhan University, Wuhan, Hubei 430060, China; ^2^Department of Plastic Surgery, Renmin Hospital of Wuhan University, Wuhan, Hubei 430060, China; ^3^Department of Hospital Infection Office, Renmin Hospital of Wuhan University, Wuhan, Hubei 430060, China

## Abstract

It has long been recognized that an increase of reactive oxygen species (ROS) can modify the cell-signaling proteins and have functional consequences, which successively mediate pathological processes such as atherosclerosis, diabetes, unchecked growth, neurodegeneration, inflammation, and aging. While numerous articles have demonstrated the impacts of ROS on various signaling pathways and clarify the mechanism of action of cell-signaling proteins, their influence on the level of intracellular ROS, and their complex interactions among multiple ROS associated signaling pathways, the systemic summary is necessary. In this review paper, we particularly focus on the pattern of the generation and homeostasis of intracellular ROS, the mechanisms and targets of ROS impacting on cell-signaling proteins (NF-*κ*B, MAPKs, Keap1-Nrf2-ARE, and PI3K-Akt), ion channels and transporters (Ca^2+^ and mPTP), and modifying protein kinase and Ubiquitination/Proteasome System.

## 1. Introduction

Reactive oxygen species (ROS), generated through a variety of extracellular and intracellular actions, have drawn attention as novel signal mediators which are involved in growth, differentiation, progression, and death of the cell [1, 2]. As a group of chemical species that include at least one oxygen atom in each molecule but display stronger reactivity than molecular oxygen, ROS comprise free radicals such as superoxide, hydroxyl radical, and singlet oxygen, as well as nonradical species such as hydrogen peroxide formed by the partial reduction of oxygen [3–5]. Oxygen free radicals are highly reactive and have the capacity to damage cellular components such as proteins, lipids, and nucleic acids. Classically, ROS were regarded as host defending molecule released by neutrophil for destructing exogenous pathogens such as bacteria; however, accumulated evidence indicates that ROS play central roles in determination of cell fate as second messengers and modifying of various signaling molecules [6–9].

It has been demonstrated that ROS have impacts on several signaling pathways and the mechanisms of how ROS act on cell-signaling proteins, how the cell-signaling proteins influence the level of intracellular ROS in turn, and if there are complex interactions between different ROS associated signaling pathways have been clarified, but the systemic summary is necessary. In this review, we focus on the pattern of the generation and homeostasis of intracellular ROS, the mechanisms and targets of ROS impacting on cell-signaling proteins, ion channels and transporters, and modifying kinases and Ubiquitination/Proteasome System.

## 2. The Homeostasis of ROS

**Figure 1:**
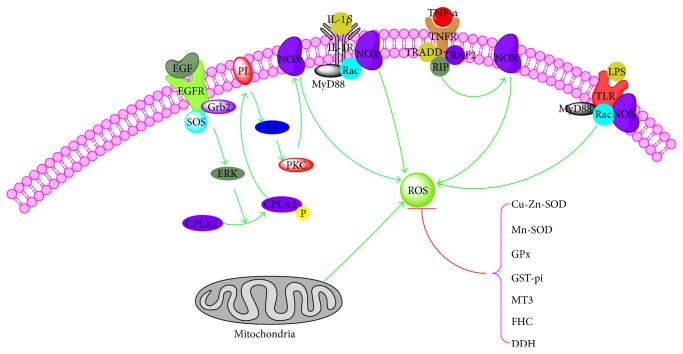
Homeostasis of intracellular reactive oxygen species. NOX, NADPH oxidases; TNF-*α*, tumor necrosis factor-*α*; EGF, epidermal growth factor; IL-1*β*, Interleukin-1*β*; SOD, superoxide dismutase; GPx, glutathione peroxidase; GST-pi, glutathione S-transferase pi; MT3, metallothionein-3; FHC, ferritin heavy chain; DDH1, dihydrodiol dehydrogenase; TNFR, tumor necrosis factor receptor; TRADD, TNFRSF1A-associated via death domain; MyD88, myeloid differentiation factor 88; TLR, Toll-like receptor; cPLA2, cytosolic phospholipases A2.

Under a physiological state, the level of cellular ROS is stable in a dynamic equilibrium, and this balance is modulated by cellular processes that produce ROS and eliminate them (Figure 1).

The resource of cellular ROS could be broadly divided into two main categories: firstly, there are those biological processes, mainly the mitochondrial oxidative metabolism, that release ROS as a byproduct, or a waste product, of various other necessary reactions and, secondly, there are those processes, in cellular response to xenobiotics, cytokines, and bacterial invasion, that generate ROS intentionally, either in molecular synthesis or in breakdown, as part of a signal transduction pathway, or as part of a cell defense mechanism [10–12]. The initial product of the mitochondrial respiratory chain is O_2_^−∙^ mainly generated by complexes I and III and could be quickly transformed into H_2_O_2_ by the enzyme superoxide dismutase (SOD) and then could be reduced to water by catalase or glutathione peroxidase [13–16]. NADPH oxidases (Nox) including Nox1 to Nox5 and Duox1 and Duox2, which are classified into three groups, according to the presence of domains in addition to the gp91phox (NOX2) domain, are another important source of cellular ROS [17, 18]: NOX1, NOX3, and NOX4 are similar in size and domain structure to NOX2, catalyzing the NADPH-dependent reduction of oxygen to form superoxide, which can react with itself to form H_2_O_2_; NOX5 is slightly different in domain structure to NOX2 but with similar process of superoxide formation; Duox1 and Duox2 contain a peroxidase-homology domain, utilizing ROS generated by the catalytic core to generate more powerful oxidant species that then oxidize extracellular substrates [19]. Meanwhile, external stimuli including tumor necrosis factor-*α* (TNF-*α*), epidermal growth factor (EGF), Interleukin-1*β* (IL-1*β*), and hypoxia and irradiation also stimulate the formation of ROS [20–24].

And, as a critical role to withstand the excessive formation of intracellular ROS, series of antioxidant proteins have been found. The main category of these antioxidant proteins is superoxide dismutases (SOD) which contain Cu-Zn-SOD (SOD1) and Mn-SOD (SOD2) [25]. SOD2, in the matrix, converts superoxide, which cannot diffuse across membranes, to H_2_O_2_ which then is reduced to water by catalase. Compared to SOD2, SOD1 mainly reduces the superoxide of intermembrane space and cytosol to H_2_O_2_. Besides, glutathione peroxidase (GPx), glutathione S-transferase pi (GST-pi), metallothionein-3 (MT3), ferritin heavy chain (FHC), and dihydrodiol dehydrogenase (DDH1 or AKR1C1) and so on also play decisive roles in the process of antioxidant [26–29].

## 3. ROS and NF-***κ***B Signaling Pathway

The transcription factor NF-*κ*B is crucial in a series of cellular processes, including immune, inflammatory response, cellular adhesion, differentiation, proliferation, autophagy, senescence, and apoptosis [30]. Likewise, the disorder of NF-*κ*B has already been confirmed to be associated with cancer, arthritis, inflammation, asthma, neurodegenerative diseases, and heart disease [31]. The family of NF-*κ*B consists of Rel (c-Rel), RelA (p65), RelB, p50/p105 (NF-*κ*B1), and p52/p100 (NF-*κ*B2). NF-*κ*B pathway may be activated by at least two distinct pathways named the canonical and noncanonical pathways. The canonical NF-*κ*B-activating pathway is triggered in response to microbial products, stress, and proinflammatory cytokines and it depends on the phosphorylation of I*κ*B-kinase (IKK) *β* and the phosphorylation and ubiquitination of I*κ*Ba and its degradation by the proteasome, and then NF-*κ*B translocates into the nucleus where it activates the transcription of target genes [32–34]. In contrast, the noncanonical NF-*κ*B-activating pathway is activated by B-cell activating factor (BAFF) [35], lymphotoxin *β* (LT*β*) [36], CD40 ligand [37], CD27 ligand [38], human T-cell leukemia virus (HTLV) [39], and Epstein-Barr virus (EBV) [40] and it relies on IKK*α* and causes activation of NF-*κ*B2/RelB complexes by inducing the proteolytic processing of the NF-*κ*B2/p100 precursor.

**Figure 2:**
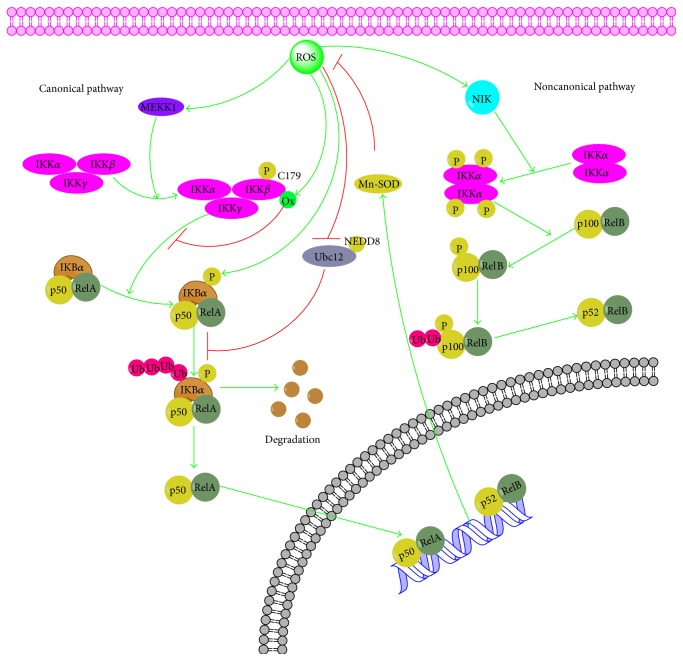
Cross talk between ROS and NF-*κ*B signaling pathway. MEKK1, mitogen-activated protein kinase kinase kinase 1; PKC, protein kinase C; NIK, NF-*κ*B inducing kinase; NEDD8, neural precursor cell expressed developmentally downregulated 8.

Recently, cumulative evidence has indicated that there is an interrelation between ROS and NF-*κ*B. Firstly, ROS influence the activation of NF-*κ*B pathway mainly by inhibiting the phosphorylation of I*κ*B*α*. A series of studies has testified that I*κ*B*α* is usually phosphorylated on serines 32 and 36 by IKK leading to its ubiquitination and degradation and exogenously added H_2_O_2_ affects the phosphorylation of I*κ*B*α* on Tyr42 or other tyrosine residues and subsequent degradation of I*κ*B*α* and activation of NF-*κ*B pathway [41, 42]. In addition, IKK is also the primary target for ROS in influencing NF-*κ*B and the S-glutathionylation of IKK*β* on cysteine 179 by ROS results in the inhibition of IKK*β* activity [43]. Then, MEKK1, the kinases upstream of IKK, may be potentially regulated by ROS. MEKK1 is a redox-sensitive kinase that could be glutathionylated at C1238 leading to its inactivation [44]. Thirdly, ROS also could disturb the ubiquitination and degradation of I*κ*B and then the activation of NF-*κ*B by inactivating Ubc12. Furthermore, NIK, the upstream kinase in the noncanonical pathway, is believed to be activated by ROS through inhibition of phosphatases and oxidation of cysteine residues [45, 46]. Meanwhile, NF-*κ*B pathway also can influence the ROS levels by increasing expression of antioxidant proteins such as Cu-Zn-SOD, Mn-SOD, GPx, GST-pi, MT3, and FHC (Figure 2).

## 4. ROS and MAPKs Signaling Pathway

The mitogen-activated protein kinase (MAPK) cascades, consisting of the extracellular signal-related kinases (ERK1/2), the c-Jun N-terminal kinases (JNK), the p38 kinase (p38), and the big MAP kinase 1 (BMK1/ERK5) pathway [47], are major intracellular signal transduction pathways that play an important role in various cellular processes such as cell growth, differentiation, development, cell cycle, survival, and cell death [48]. Similarly, ERK, JNK, p38, and BMK1 are all serine/threonine kinases that are directed by a proline residue. Along with the pathways in which these four MAP kinases are activated share similarity by extracellular or intracellular stimuli, a MAP kinase kinase kinase (MAPKKK) is activated and then phosphorylating and activating a MAP kinase kinase (MAPKK) and the MAPKK phosphorylating and activating a MAP kinase (MAPK) and activated MAPKs phosphorylate various substrate proteins, resulting in regulation of various cellular activities [49–51].

The ERK pathway is activated mainly by growth factors (epidermal growth factor, EGF, and platelet-derived growth factor, PDGF) and cytokines (IL-1*β* and TNF-*α*), and its activation is related to the stimulation of tyrosine kinase receptors [52, 53]. When these receptors of growth factors and cytokines are bound with their ligands, the GDP bound Ras is converted to GTP that in turn activates Ras. Subsequently activated Ras recruits cytoplasmic Raf (MAPKKK) to the cell membrane for activation. Activated Raf phosphorylates MEK1/2 (MAPKK), which then phosphorylates ERK1/2 (MAPK) that translocate to the nucleus and activates several transcription factors [54, 55]. ROS have been shown to activate the receptors of EGF and PDGF, though without corresponding ligands, which can stimulate Ras and the subsequent activation of ERK pathway [56, 57]. In addition, it has been demonstrated that ROS generated by commensal bacteria inactivated dual-specific phosphatase 3 (DUSP3) by oxidation on Cys-124 results in ERK activation [58]. Meanwhile, in some cells, treatment with H_2_O_2_ leads to the phosphorylation and activation of phospholipase C- (PLC-) gamma which results in the generation of inositol trisphosphate (IP3) and diacylglycerol (DAG) [59]. IP3 could increase the intracellular calcium by inducing the release of calcium from intracellular stores that can mediate activation of ERK pathway and generation of DAG and increases in intracellular calcium which results in the activation of several forms of protein kinase C (PKC) leading to Ras and Raf activation [60, 61].

**Figure 3:**
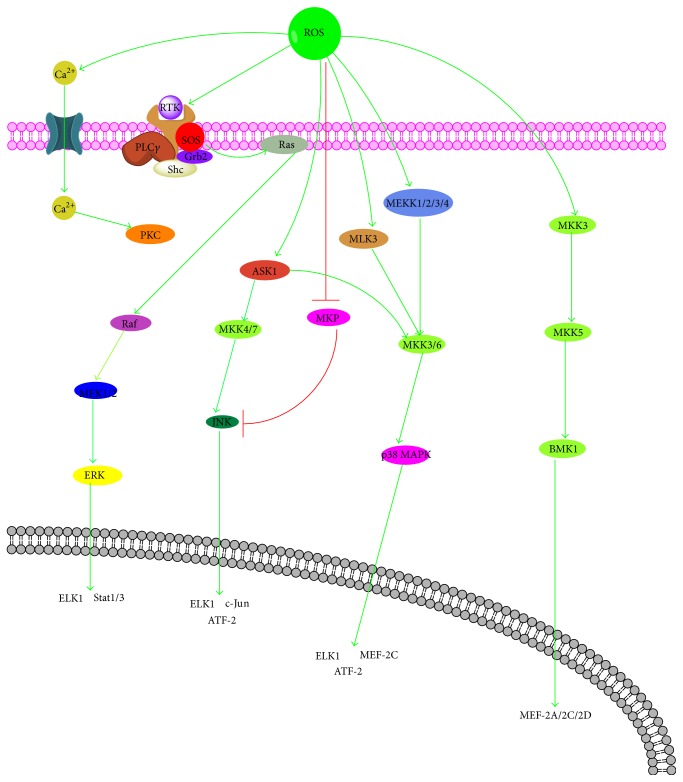
Cross talk between ROS and MAPKs signaling pathway. MAPK, mitogen-activated protein kinase; ERK, extracellular signal-related kinases; JNK, c-Jun N-terminal kinases; p38, p38 kinase; BMK1/ERK5, big MAP kinase 1; MAPKKK, MAP kinase kinase kinase; MAPKK, MAP kinase kinase; MAPK, MAP kinase; PLC, phospholipase C; IP3, inositol trisphosphate; DAG, diacylglycerol.

The JNK pathway is activated by environmental stress (oxidative stress) and cytokines (tumor necrosis factor, TNF, and FAS) and involves a kinase cascade similar to the ERK pathway with a MAPKKK activating a MAPKK and the MAPKK subsequently phosphorylating JNK on critical threonine and tyrosine residues resulting in the activation of JNK; afterwards JNK translocate to the nucleus and regulate the activity of multiple transcription factors. The MAPKKK in JNK pathway includes MEKK1, MEKK2, MEKK3, and MEKK4, MLK, and ASK1 and MAPKK contain MKK4, MKK3, MKK6, and MKK7 [62, 63]. ROS could act on TRX and glutaredoxin, a kind of redox-sensitive proteins, to dissociate from ASK-1 for its activation, resulting in the activation of JNK [64]. Also, ROS could trigger the detachment of JNK from glutathione S-transferase pi (GSTp), which can interact with JNK to suppress its activation, thereby facilitating JNK activation [65]. ROS could be able to allow ASK1 to be oligomerized and autophosphorylated and become activated by oxidizing thioredoxin, which inhibits the activation of ASK1 via binding to the N-terminal of ASK1 [66]. TNF receptor-associated JNK activation is thought to be mediated in part by oxygen radicals because superoxide anion and lipid peroxide-scavengers inhibit JNK activation. Furthermore, it is possible that low levels of ROS intermediates leave phosphatase activity intact, leading to a transient activation of JNK. Higher levels of ROS may activate JNK pathway and inactivate the phosphatases resulting in a prolonged activation of JNK (Figure 3).

The p38 pathway is activated by extracellular stresses, growth factor, and cytokines, such as tumor necrosis factor-a (TNF-a) and IL-1*β*. The TNF receptors switch on the p38 pathway via the activation of cdc42, whereas growth factor receptors switch on the p38 pathway by the sequential activation of Ras and Rac1 [67]. Small G-proteins Rac1 and cdc42 activate ASK1, MLK3, and MLK3 that directly activate MKK3 and MKK6 which phosphorylates p38 on both tyrosine and threonine residue resulting in the activation of p38 pathway [68, 69]. Some initial proteins, such as ASK-1, in the JNK pathway, are also involved in the activation of the p38 pathway. Oxidative stress directly or indirectly affects ASK1, MEKK1, MEKK2, MEKK3, MEKK4, and MLK3 and subsequently activates p38 pathway (Figure 3).

The BMK1 (also known as ERK5) pathway, which has been involved in cell survival, antiapoptotic signaling, angiogenesis, cell motility, differentiation, and cell proliferation, is one of the least studied members of the MAPK family [70]. Oxidative stress (H_2_O_2_) could influence BMK1 pathway by activating MEKK2 and MEKK3 directly. Then MEK5 and BMK1 are activated sequentially and BMK1 acts on its downstream targets including Mef2C, c-Myc, and possibly Nrf2 (Figure 3).

## 5. ROS and Keap1-Nrf2-ARE Signaling Pathway

Another signaling pathway, Keap1-Nrf2-ARE, performs critical role in maintaining the cellular redox balance and metabolism and inducing an adaptive response for oxidative stress that can otherwise lead to many inflammatory diseases including cancer, Alzheimer's disease (AD), Parkinson's disease (PD), and diabetes. This pathway consists of three main cellular components: Kelch-like ECH-associated protein 1 (Keap1), nuclear factor erythroid 2-related factor 2 (Nrf2), and antioxidant response elements (ARE) [71–76]. Under normal physiological conditions, Keap1, which is also called an inhibitor of Nrf2 (INrf2), is associated with Nrf2 (the majority of which resides in the cytoplasm) and recruits and interacts with the cullin-3 E3-ubiquitin ligase (Cul3) [77]. And the ubiquitination of Nrf2 is stimulated that targeted Nrf2 for degradation by the 26S proteasome (more related information has been provided in “ROS and Ubiquitination/Proteasome System” section) [78].

**Figure 4:**
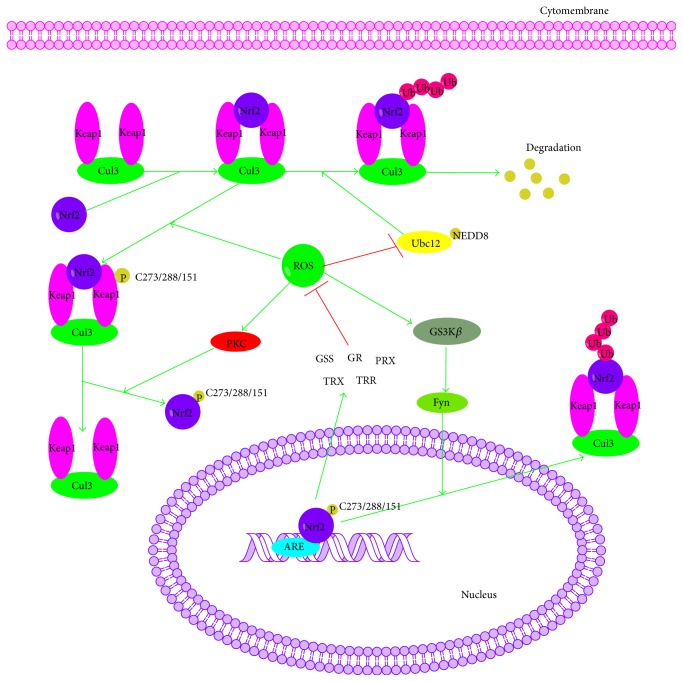
Cross talk between ROS and Keap1-Nrf2-ARE signaling pathway. Keap1, Kelch-like ECH-associated protein 1; Nrf2, nuclear factor erythroid 2-related factor 2; ARE, antioxidant response elements; Cul3, cullin-3 E3-ubiquitin ligase; GSK3*β*, glycogen synthase kinase 3; Ubc, E2-ubiquitin conjugating enzyme.

However, under oxidizing conditions, the increased level of intracellular ROS promotes the dissociation of Nrf2 and Keap1, either by the oxidization of key reactive cysteine residues (Cys273, Cys288, and Cys151) that govern Keap1 activity or via the activation of kinases, such as protein kinase C (PKC), MAPK, phosphatidylinositide 3-kinases (PI3Ks), and protein kinase-like endoplasmic reticulum kinase (PERK) that phosphorylate Nrf2 [79–81]. After that the dissociated Nrf2 is transferred to the nucleus where it dimerizes with members of another b-zip family, the small Maf proteins (Maf-F, Maf-G, and Maf-K), binds to ARE of phase II genes, and translates detoxification enzymes such as glutathione synthetase (GSS), glutathione reductase (GR), Gpx, thioredoxin (TRX), thioredoxin reductase (TRR), and peroxiredoxin (PRX) to prevent the oxidative stress [73, 82]. Meanwhile, oxidative stress activates GSK3*β* leading to nuclear import of Src kinases such as Src, Yes, Fyn, and Fgr, which phosphorylates Nrf2 (Tyr568) followed by the nuclear export with Keap1 and degradation of Nrf2 [83, 84] (Figure 4).

## 6. ROS and PI3K-Akt Signaling Pathway

The phosphoinositide-3-kinase- (PI3K-) Akt pathway has been involved in many critical cellular functions, including protein synthesis, cell cycle progression, proliferation, apoptosis, autophagy, and drug resistance in response to growth factor (EGF, PDGF, NGF, and VEGF), hormone (prostaglandin, PGE_2_), and cytokine (IL-17, IL-6, and IL-2) stimulation [85–87]. The binding of growth factor to its receptors directly stimulates class 1A PI3Ks bound via their regulatory subunit or adapter molecules such as the insulin receptor substrate (IRS) proteins, which subsequently triggers the activation of PI3K. Afterwards, the activated PI3K catalyzes the synthesis of phosphatidylinositol 3,4,5-triphosphate (PIP3), from phosphatidylinositol 4,5-bisphosphate (PIP2) [88]. The membranal PIP3, a signaling molecule, recruits and activates proteins that contain the pleckstrin homology (PH) domain such as the phosphoinositide-dependent protein kinase (PDK) and protein kinase B (Akt) serine/threonine kinases and the activation of PDK and Akt successively promotes the activation and transcription of their target genes (GSK3, FOXO, BAD, mTOR1, and p53) [89–92].

**Figure 5:**
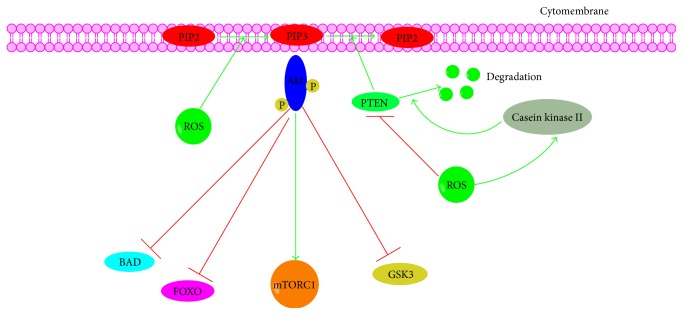
Cross talk between ROS and PI3K-Akt signaling pathway. PI3K, phosphoinositide-3-kinase; Akt, protein kinase B; PTEN, phosphatase and tensin homolog; FOXO, forkhead box protein O; mTOR1, mechanistic target of rapamycin 1.

ROS not only activate PI3K directly to amplify its downstream signaling but also concurrently inactivate phosphatase and tensin homolog (PTEN), which negatively regulates the synthesis of PIP3 and thereby inhibits the activation of Akt, via oxidizing cysteine residues within the active center [93]. In addition, ROS is able to promote the phosphorylation by casein kinase II on PTEN which urges PTEN to enter the proteolytic degradation pathway [93]. Furthermore, protein phosphatase 2A (PP2A), which might be deactivated by ROS, could inhibit Akt/PKB. However, it seems that, at lower levels, ROS oxidize the disulfide bridges in Akt/PKB, leading to the association of Akt/PKB with PP2A and thus short-term activation of Akt/PKB [46, 94, 95] (Figure 5).

## 7. Cross Talk between ROS and Ca^**2+**^

**Figure 6:**
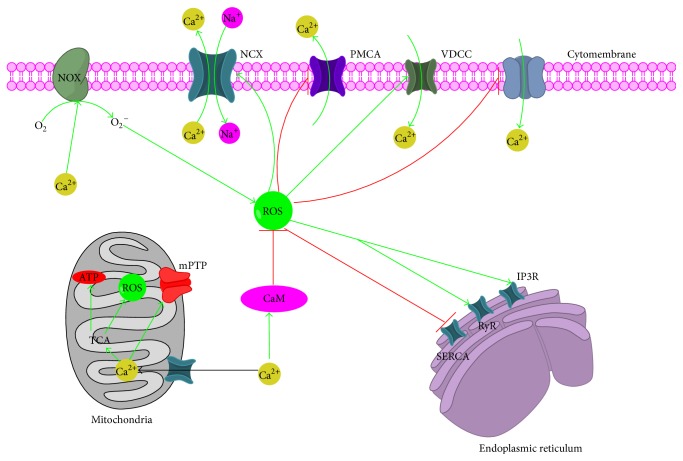
Cross talk between ROS and Ca^2+^. IP3R, inositol 1,4,5-trisphosphate receptor; RyR, the ryanodine receptor; VDCC, voltage-dependent Ca^2+^ channels; SOC, store-operated Ca^2+^ channel; SERCA, sarcoplasmic/endoplasmic reticulum Ca^2+^ ATPase; PMCA, plasma membrane Ca^2+^ ATPase; MCU, mitochondrial Ca^2+^ uniporter; TCA cycle, tricarboxylic acid cycle; NCX, Na^+^/ Ca^2+^ exchanger.

In eukaryotic cells, Ca^2+^ is one of the most versatile signals involved in the control cellular processes and functions, such as contraction, secretion, metabolism, gene expression, cell survival, and cell death [96, 97]. Cytosolic Ca^2+^ concentration ([Ca^2+^]_c_) is determined by a dynamic balance between the mechanisms that pour Ca^2+^ into the cytoplasm, including Ca^2+^ influx from the extracellular medium and intracellular stores such as endoplasmic reticulum (ER) or sarcoplasmic reticulum (SR), and those processes that remove it out, involving Ca^2+^ efflux across the plasma membrane and sequestration into mitochondria [98, 99]. The uptake mechanisms of Ca^2+^ into the cytoplasm refer to the inositol 1,4,5-trisphosphate receptor (IP_3_R), the ryanodine receptor (RyR), and the nicotinic acid-adenine dinucleotide phosphate (NAADP) that are responsible for Ca^2+^ release from ER and SR, as well as voltage-dependent Ca^2+^ channels (VDCC) and store-operated Ca^2+^ channel (SOC), which are in charge of Ca^2+^ influx from extracellular matrix [100–102]. Meanwhile, the mechanisms of removing Ca^2+^ are determined by the plasma membrane Ca^2+^ ATPase (PMCA), which mediates Ca^2+^ extrusion across the plasma membrane into the cytoplasm, the sarcoplasmic/endoplasmic reticulum Ca^2+^ ATPase (SERCA), which reintroduces Ca^2+^ into the ER/SR, Na^+^/Ca^2+^ exchanger (NCX) that involves the clearance of Ca^2+^ through its exchange by Na^+^, and the mitochondrial Ca^2+^ uniporter (MCU) that transports Ca^2+^ into the mitochondria [103, 104]. Recent studies have demonstrated that the ROS and Ca^2+^ signaling systems influence each other in various ways (Figure 6).

Numerous evidences indicate that intracellular Ca^2+^ modulates both ROS generation and ROS clearance processes and thereby shift the redox state to either more oxidized or reduced state. The primary role of Ca^2+^ is the promotion of ATP synthesis and ROS generation in mitochondria via stimulating the Krebs cycle enzymes and oxidative phosphorylation [105]. The mitochondrial respiratory chain provides the main source of physiological ROS production (O_2_^−∙^), which is either converted to H_2_O_2_ by spontaneous dismutation or catalyzed by SOD. Mitochondrial Ca^2+^ could activate three dehydrogenases of the TCA cycle (pyruvate dehydrogenase, isocitrate dehydrogenase, and oxoglutarate dehydrogenase), the ATP synthase (complex V), and the adenine nucleotide translocase and then increase the generation of ROS [106–108]. Along with that, Ca^2+^ regulates multiple extramitochondrial ROS generating enzymes, including NOX [109] and nitric oxide synthase (NOS) [110], both in physiological and in pathological processes. Meanwhile, Ca^2+^ modulates ROS clearance processes via regulating the antioxidant defense system: on one hand, Ca^2+^ can directly activate antioxidant enzymes (catalase and GSH reductase), increase the level of SOD, and induce mitochondrial GSH release early in Ca^2+^-induced mitochondrial permeability transition pore (mPTP) opening; on the other hand, calmodulin (CaM), ubiquitous Ca^2+^-binding protein, could activate catalases in the presence of Ca^2+^ and downregulates H_2_O_2_ levels [111–113].

Furthermore, ROS also influences Ca^2+^ signaling via oxidizing Cys thiol of Ca^2+^ channels/pumps/exchangers involving RyR, IP_3_R, SERCA, PMCA, and NCX. RyR/IP_3_R, as well as many of the regulatory proteins that form complex with the RyR/IP_3_R, contains multiple reactive Cys thiols that influence channel gating or assembly [114]. Thiol oxidation of RyR/IP_3_R by ROS in general increases channel activity and thereby promotes Ca^2+^ efflux via enhancing intersubunit binding and preventing the binding of the negative regulator calmodulin to the receptor [115]. As with RyR/IP_3_R, SERCA pumps also contain numerous free Cys residues which are oxidized by ROS in the context of oxidation state which inhibits the activity of SERCA and decreases Ca^2+^ influx from the cytoplasm to ER [116]. Additionally, although PMCA is a slower pump than SERCA, it can be reversibly inactivated by ROS by altering the Tyr^589^, Met^622^, and Met^831^ residues [117]. And ROS both stimulate and decrease NCX activity: H_2_O_2_ generated from the xanthine/xanthine oxidase system enhances NCX activity and oxidants from hypoxanthine/xanthine oxidase depress NCX activity. Moreover, ROS also alter the activity of VDCC, especially the activity of L-type Ca^2+^ channels, which has been associated with the oxidation of -SH groups resulting in altered Ca^2+^ entry in the cytoplasm [118].

## 8. ROS and mPTP

**Figure 7:**
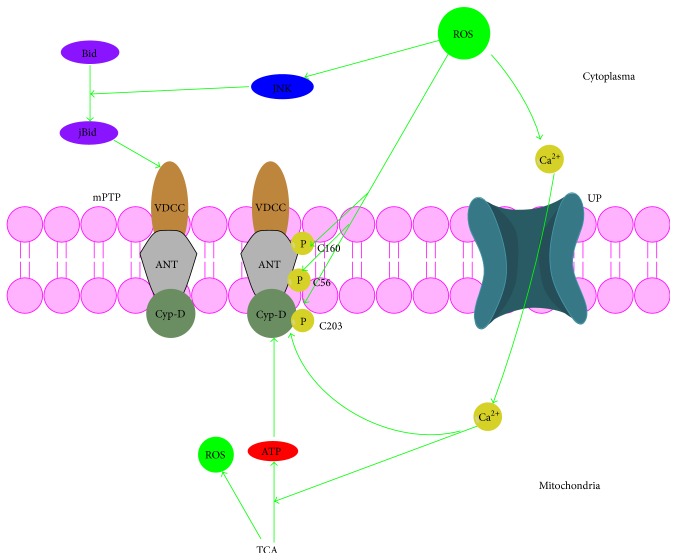
Cross talk between ROS and mPTP. VDAC, voltage-dependent anion channel; ANT, adenine nucleotide translocator; Cyp-D, cyclophilin-D.

Several studies, lasting for decades, have showed that mPTP, a large, nonspecific channel spanning the inner mitochondrial membrane (IMM) and outer mitochondrial membrane (OMM) [119, 120], mediates the lethal permeability changes that initiate mitochondrial-driven death. Hitherto, the putative components include the voltage-dependent anion channel (VDAC) or porin, localized in the OMM; the adenine nucleotide translocator (ANT) in the IMM; the peripheral benzodiazepine receptor and the Bcl-2 family proteins; the hexokinase bound to porin; the cyclophilin-D (Cyp-D), a regulatory element in the matrix; glycogen synthase kinase-3b (GSK-3b); and cytochrome c [121–123]. It has been described that when mPTP opens by the activation of various signals, mitochondrial permeability is changed which dissipates the proton electrochemical gradient (ΔΨ*m*), which drives multiple mitochondrial functions, leading to ATP depletion, further reactive oxygen species production, and ultimately swelling and rupture of the organelle. This in turn releases proapoptotic proteins: cytochrome c (Cyt C) [124] binds to apoptotic protease activating factor-1 (Apaf1) and then forms apoptosome that activates the caspase-9 and caspase-3 protease system and induces apoptosis, Smac/DIABLO [125] activates caspases by sequestering caspase-inhibitory proteins, and endonuclease-G (endoG) [126] mediates DNA fragmentation. Factors like the changes of intracellular Ca^2+^, the level of ATP/ADP, the release of Cyt C, regulation in mitochondrial morphology, and ROS generation often influence the mPTP opening [127] (Figure 7).

The mechanism of ROS mediating the mPTP formation involves several pathways. Firstly, ROS directly modulate mPTP opening by oxidizing four different sites: Cys^160^ of ANT, regulated by glutathione oxidation and protected by low concentration of N-ethylmaleimide (NEM) or monobromobimane [128]; Cys56 of ANT, sensitive to the redox state of the matricial pyridine nucleotides perhaps with the mediation of thioredoxin or lipoamide and also protected by NEM, not by monobromobimane [129]; external thiol groups (SH), promoting PTP opening by reaction with NEM or copper-orthophenanthroline; and Cys^203^ of Cyp-D, S-glutathionylation of which prevents Cyp-D binding to ANT which blocks MPT [130]. Besides, ROS indirectly modulate the opening of mPTP via increasing the mitochondrial Ca^2+^ concentration ([Ca^2+^]_m_): ROS promotes Ca^2+^ efflux from ER/SR to cytoplasm and from cytoplasm to mitochondria. The increase of Ca^2+^ concentration in turn favors ATP production and ROS generation during oxidative phosphorylation and promotes the opening of mPTP [131, 132]. In addition, ROS also may translocate Bid to jBid via activating the JNK pathway, which leads to the opening of mPTP [133–135].

## 9. ROS and Protein Kinase

**Figure 8:**
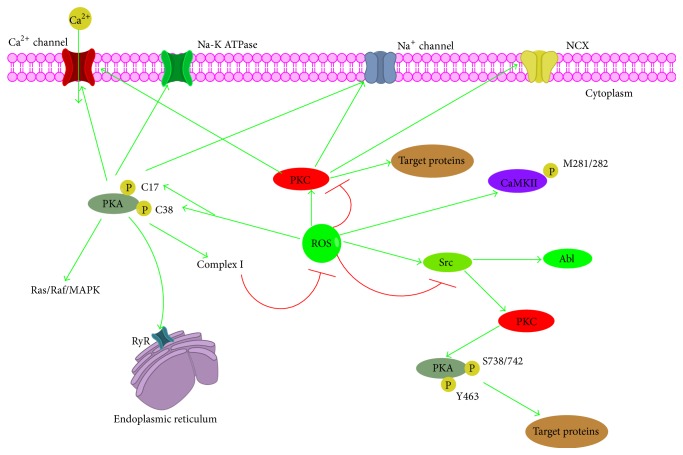
Cross talk between ROS and protein kinase. CaMKII, calcium/calmodulin-dependent protein kinase II; RyR, the ryanodine receptor.

Recently, it is becoming increasingly apparent that, like physiological second messengers in signal transduction, ROS function in various cellular processes via oxidating sulfhydryl (SH) groups of cysteine residues in protein kinases including protein kinase A (PKA) [136], protein kinase C (PKC) [137], protein kinase D (PKD) [138], receptor tyrosine kinase (RTK) [139], and Ca/calmodulin independent protein kinase II (CaMKII) [140] and then activated protein kinases phosphorylate their target proteins which are involved in different cellular signaling mechanisms (Figure 8).

PKA, also called cAMP-dependent protein kinase A, is organized as tetramers comprising two catalytic and two regulatory subunits. The activation of PKA can occur by binding of two molecules of cAMP to each regulatory subunit and then this activated PKA phosphorylates its targeting proteins, including RyR and L-type Ca^2+^ channel and phospholamban (PLN) [141, 142]. Recently, it has been shown that type I regulatory subunit I of PKA is subjected to oxidation by ROS on Cys 17 and 38, which leads to the intersubunit disulfide bond formation (between two regulatory subunits) and dissociation of the PKA holoenzyme complex. And the translocation (from cytosol to membrane and myofilaments) and activation of type I PKA result in increased cellular contractility without elevations in cAMP [143]. Meanwhile, not only do ROS influence the phosphorylation of PKA, but phosphorylation of PKA also has an impact on the ROS homeostasis. In mammalian cells, the cAMP/PKA pathway regulates the expression, assembly, and catalytic activity of complex I of the mitochondrial respiratory chain and subsequently determines the synthesis and accumulation of ROS [144, 145].

Protein kinase C (PKC), containing four homologous domains termed C1, C2, C3, and C4, is a superfamily of structurally correlated serine-threonine kinases that catalyze numerous critical biochemical reactions, like cellular responses, gene expression, cell proliferation, survival, and migration [146]. In an inactive state, PKC is loosely associated with membrane lipids and chiefly isolated in the cytosolic fraction, whereas activation of PKC increases the affinity of the enzyme for membrane lipids and consequently stabilizes its membrane association, which causes a conformational change to a catalytically competent form of PKC [147–149]. Both the regulatory and catalytic domains of PKC contain cysteine-rich regions, thus making it a highly susceptible direct target for redox regulation. Oxidants play a dual role in both stimulation and inactivation of PKC with relation to the concentration: higher doses of oxidants react with catalytically important cysteine residues inactivating PKC; however, low doses induce stimulation of PKC activity. It has been found that H_2_O_2_ stimulated the activation of tyrosine kinases and was able to indirectly regulate the tyrosine phosphorylation of PKC-*δ* at residues 512 and 523 [150].

PKD isoforms (PKD1, PKD2, and PKD3), the effectors of diacylglycerol (DAG), and protein kinase c (PKC) effectors have been described as vital regulators of diverse cellular pathways and mediate the actions of growth factors, neurotransmitters, hormones, and other stimuli that activate PLC*β* and PLC*γ* [151–153]. The binding of the corresponding ligand to G-protein coupled receptors (GPCRs) or tyrosine kinase receptors activates PLC*β* and PLC*γ*. Then PLC*β* cleave PI (4, 5) P2 that generates DAG and IP3. Subsequently, membranal DAG binds to and activates PKC and recruits PKD, which then is phosphorylated and activated by PKC on Ser^744^ and Ser^748^ residues [154, 155]. ROS influence the activation of PKD in a various manner: ROS trigger PLD1 and phosphatidic acid phosphatase- (PAP-) catalyzed DAG synthesis and concomitant recruitment of PKD1 and PKC*δ* at the outer mitochondrial membrane [156]; ROS promotes phosphorylation of PKD on its Tyr^93^ residue by Src that creates a binding site for the PKC*δ* C2 domain which facilitates the binding between PKC*δ* with PKD and the activation of PKD [157]; ROS also could lead to the activation of PKD via the phosphorylation at Tyr^463^ residue by the tyrosine kinase Abl. Additionally, expression of mitochondrial Mn-SOD induced by PKD1-NF-*κ*B signaling removes toxic ROS [158].

Moreover, the activation of RTK and CaMKII could be affected by the level of intracellular ROS. The oxidation on Met^281^ and Met^282^ residues in the regulatory domain results in the activation of CaMKII [159]. And RTKs such as the insulin receptor, EGFR, platelet-derived growth factor receptor (PGFR), and c-Ret have all been reported to undergo direct oxidation on their cysteine residue.

## 10. ROS and Ubiquitination/Proteasome System

Ubiquitination/Proteasome System (UPS) includes four components: proteasome, ubiquitin, the ubiquitination machinery, and the deubiquitinases (DUBs) [160]. UPS play indispensable roles in variety of biological processes such as regulation of the cell cycle, inflammatory responses, immune response, protein misfolding, and endoplasmic reticulum-associated degradation of proteins. Initially, ubiquitin gets activated by an ATP-dependent E1 ubiquitin-activating enzyme which results in the transient adenylation of ubiquitin and the transference of ubiquitin from E1 to a cysteine residue of E2-ubiquitin conjugating enzyme (Ubc); then E3 transfers ubiquitin from E2-ubiquitin to the lysine residue of a substrate protein by catalyzing the peptidyl bond formation between ubiquitin and the target protein and subsequently the elongation of the polyubiquitin chain which transfers the client protein to the proteasome for degradation through specific proteolytic activities [161–163]. Concurrently, DUBs can remove ubiquitin from substrates and disassemble polyubiquitin chains which may lead to protein stabilization [164].

**Figure 9:**
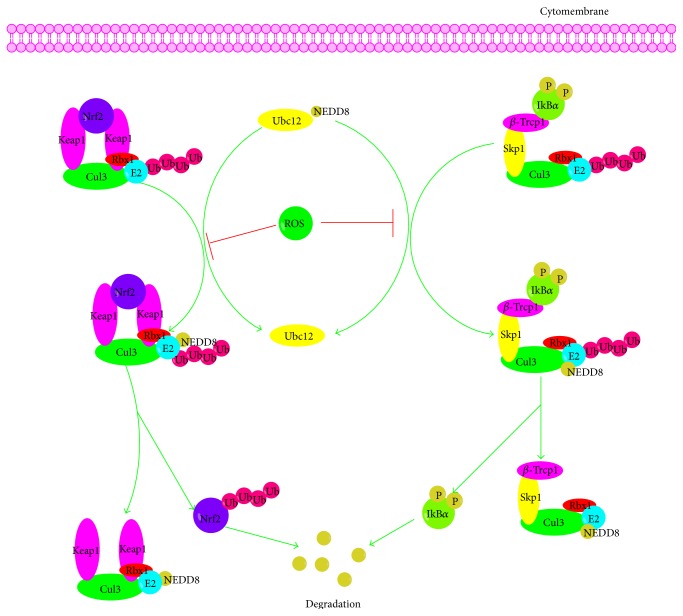
Regulation of Ubiquitination/Proteasome System by ROS. Ubc, E2-ubiquitin conjugating enzyme.

Recently, an increasing number of studies have documented the interactions between ROS and UPS [165–168]. The susceptibility of the UPP to oxidative stress may have been anticipated, because E1, E2, some E3 enzymes, and DUBs have a cysteine residue, which are sensitive to ROS, in their active sites (Figure 9). The rapid depletion of reduced glutathione (GSH) and improvement of the levels of oxidized glutathione (GSSG) upon exposure to oxidative stress result in the oxidation of cysteine residues in the active sites of E1 and E2 and the generation of mixed disulfide bonds which blocks their binding to ubiquitin [169, 170]. It has also been reported that bacteria elicit ROS generation in epithelial cells that inactivate the Ubc12 enzyme, preventing the neddylation of cullin-1. Unneddylated cullin in E3-SCF^*β*-TrCP^ complex renders it unable to carry out ubiquitination and is thus making it inactive [171]. Additionally, numerous reports have suggested that Kelch-like ECH-associated protein-1 (Keap1), a substrate adaptor protein for a cullin-3 E3-ubiquitin ligase (Cul3)/Ring-Box1 (Rbx1) dependent complex, plays a critical role in the ubiquitination and degradation of Nrf2, IKK*β*, and Bcl-2/Bcl-xL, also being disturbed by ROS via modifying the reactive cysteines (Cys273, Cys288, and Cys151) and then inducing a conformational change that leads to the release of Nrf2, IKK*β*, and Bcl-2/Bcl-xL from Keap1 and the suspending of their ubiquitination and degradation [172–174]. Meanwhile, the proteasome is also a target of oxidative stress and the 26S proteasome was more susceptible than the 20S proteasome to oxidative inactivation [175].

In turn, UPS regulates cellular redox status via the degradation of Nrf2 and the activation of NF-*κ*B and both could mediate the level of ROS by their downstream antioxidative proteins [176]. In addition, accumulating evidences made it evident that the UPS plays essential roles in regulating mitochondrial processes: oxidative phosphorylation, TCA cycle, and mitochondrial dynamics which subsequently regulate ROS generation [177–179].

## 11. Conclusions

It has been clearly demonstrated that redox equilibrium plays pivotal roles in cells' physiological and pathological events due to ROS's ability to activate or deactivate a variety of receptors, proteins, ions, and other signaling molecules. When the redox equilibrium is disturbed due to the excessive accumulation or depletion of ROS, many cellular signaling pathways are influenced which confers to the cellular dysfunction and subsequently the development of various pathologies. Therefore, unveiling the mechanisms of ROS regulating redox-associated signaling pathways is essential in providing relevant targets in order to develop innovative and effective therapeutic strategies. However, due to numerous signaling pathways which are sensitive to ROS and the high degree of complexity in simultaneous actions of ROS, even though we have learnt much about the mechanisms by which ROS influences signaling, in particular, the interactions between different ROS associated signaling pathways are yet to be elucidated.
